# Compound steep-pulse irreversible electroporation for prostate ablation in Beagle dogs: parameter evaluation and short-term safety

**DOI:** 10.3389/fruro.2026.1721078

**Published:** 2026-09-17

**Authors:** Zhijin Chen, Linli Zhao, Zhenhua Zhang, Zhongbao Luo

**Affiliations:** 1 Department of Laboratory Medicine, Shanghai Municipal Hospital of Traditional Chinese Medicine, Shanghai University of Traditional Chinese Medicine, Shanghai, China; 2 Shanghai Nortion Medical Technology Co. Ltd., Shanghai, China

**Keywords:** ablation, Beagle dogs, compound steep pulse, irreversible electroporation, prostate gland

## Abstract

**Introduction:**

This study aimed to evaluate the ablation performance and short-term safety of compound steep-pulse irreversible electroporation (CSP-IRE) and to refine treatment parameters for prostate ablation in a canine model.

**Methods:**

Twenty healthy male Beagles were included in a sequential, stepwise parameter-optimization experiment. Single-pulse and compound-pulse protocols were compared, followed by evaluation of electric-field parameters, interelectrode spacing, and electrode insertion depth. Ablation area was assessed histopathologically, while procedure-related muscle contraction and perioperative clinical and laboratory findings were evaluated.

**Results:**

The compound-pulse protocol produced a larger ablation area than the 5-μs and 50-μs single-pulse protocols (307.27 vs 39.30 and 64.44 mm^2^, respectively). Mean ablation areas in the low-, medium-, and high-parameter groups were 39.74 ± 43.45, 175.46 ± 94.08, and 228.49 ± 41.26 mm^2^, respectively. The medium- and high-parameter groups produced significantly larger ablation areas than the low-parameter group (P < 0.05), with no significant difference between the medium- and high-parameter groups. However, the high-parameter group induced greater muscle contraction than the medium-parameter group (P < 0.05). Mean ablation areas at interelectrode spacings of 10, 14, and 18 mm were 83.93 ± 25.31, 226.25 ± 108.25, and 216.20 ± 64.17 mm^2^, respectively. Increasing the insertion depth from 10 to 20 mm resulted in only a modest increase in ablation area (83.93 ± 25.31 vs 90.10 ± 32.92 mm^2^). No serious adverse events were observed during the 7-day follow-up, and no clinically relevant changes were detected in erythrocyte, hemoglobin, or platelet measures, although transient leukocytosis occurred.

**Discussion:**

The compound-pulse protocol demonstrated greater ablation efficacy than the single-pulse protocols. The medium-parameter protocol provided a favorable balance between ablation area and procedure-related muscle contraction. These findings support the short-term feasibility of CSP-IRE and provide a basis for further parameter refinement and validation.

## 1 Introduction

Benign prostatic hyperplasia (BPH) is a common disease in older men. Men over 50 years of age have a 50% chance of developing BPH, and men over 80 years old have an 80% chance (1). The main clinical manifestations are lower urinary tract symptoms represented by frequent urination and difficulty in urination (2). Currently, the treatment of BPH includes oral medication and surgery (3). The development of BPH cannot be stopped at its source by medication, and long-term oral maintenance treatment is necessary for big prostatic enlargements. Surgery is frequently accompanied by a variety of postoperative complications (4, 5). Despite the emergence of plasma prostatectomy and various laser-based prostate surgeries in recent years, they have not changed their basic surgical operating concepts. Still, they cannot completely circumvent the common problems associated with the transurethral surgeries mentioned above (6, 7). With the development of technology, a growing number of novel approaches are opening up new avenues for treating BPH.

Irreversible electroporation (IRE) is a focal tumor ablation technique that employs high-voltage, low-energy electrical pulses delivered between needle electrodes to induce permanent nanopore formation in the cell membrane (8, 9). Unlike conventional thermal ablation modalities, tissue destruction by IRE is primarily mediated through irreversible disruption of membrane integrity, leading to loss of cellular homeostasis and subsequent apoptotic or necrotic cell death, rather than heat-induced protein denaturation or coagulative necrosis (10–12). Although transient Joule heating may occur during pulse delivery, extensive experimental, translational, and clinical evidence has demonstrated that these temperature elevations are limited and insufficient to account for the observed ablation patterns, supporting the classification of IRE as a non-thermal ablation modality (13–16). This non-thermal mechanism allows preservation of the extracellular matrix, blood vessels, and neural structures within and adjacent to the ablation zone, thereby minimizing collateral damage to surrounding healthy tissues (9). Owing to these unique biophysical characteristics, IRE has been increasingly applied in the clinical treatment of tumors in organs such as the liver, pancreas, and prostate, with favorable oncological outcomes and low rates of recurrence and metastasis (17–19). Building upon this non-thermal electroporation platform, recent technological advances have further extended IRE toward bipolar and high-frequency pulse delivery strategies, aiming to improve ablation controllability while reducing procedure-related neuromuscular stimulation.

High-frequency irreversible electroporation (H-FIRE), which delivers ultra-short bipolar electrical pulses, has been developed to address key limitations of conventional unipolar IRE, particularly procedure-related muscle contractions, while maintaining non-thermal ablation efficacy (20). Preclinical studies have demonstrated that bipolar high-frequency pulse bursts can induce effective irreversible electroporation with minimal neuromuscular stimulation, and subsequent large-animal experiments have confirmed their safety and feasibility *in vivo (*21–23). Importantly, bipolar high-frequency pulse protocols have also been evaluated in early clinical studies of prostate ablation, showing effective lesion formation with reduced muscle contraction compared with conventional IRE (15). More recently, animal studies using canine prostate models further established the feasibility and safety of H-FIRE across a range of electric field strengths and electrode configurations, thereby defining an initial parameter window for prostate ablation (24). However, existing studies have predominantly relied on fixed waveform structures or single pulse-width paradigms and have focused on feasibility rather than optimization. Whether compound steep-pulse protocols that integrate multiple pulse widths and electric field strengths within a single ablation sequence can provide additional advantages over conventional single-pulse H-FIRE schemes remains insufficiently explored, particularly in large-animal prostate models.

Based on the principle of IRE, our group has developed a therapeutic device that generates highly heavy-frequency bipolar compound steep pulses. Its bipolar pulses make the extracellular stimulus that induces action potentials always fall in the non-responsive phase of the nerve cells, which greatly improves the threshold of action potential occurrence of the nerve cells, and significantly reduces the intensity and number of muscle contractions, thus reducing the pain and discomfort of the patients (15). The device has passed the registration test at the Shanghai Testing and Inspection Institute for Medical Devices and is qualified for clinical trials. Electric field strength and pulse width are important parameters for ablation with the compound steep pulse therapeutic apparatus. At different electric field strengths and pulse widths the cell membrane produces different permeability, the degree of damage to the cells will be different, and thus the ablation effect on the lesion will be different (25).

In this study, we established a model for ablating the prostate gland in Beagle dogs to investigate compound steep-pulse irreversible electroporation (CSP-IRE) in a large-animal setting. We evaluated the effects of different pulse and electrode parameter configurations on ablation area and assessed procedure-related responses and short-term safety. The study aimed to characterize the ablation performance of compound steep-pulse protocols and provide experimental evidence for parameter refinement and further investigation in prostate ablation.

## 2 Methods

### 2.1 Experimental animals

Twenty healthy male purebred Beagles with a mean body weight of approximately 13 kg were used in this study. Each animal provided two prostate lobes (left and right) available for treatment allocation, resulting in a total of 40 prostate lobes for the predefined experimental interventions. The animals were housed at the Songjiang Experimental Animal Center of Shanghai Jiao Tong University Agriculture and Life Science Experimental Training Company under controlled environmental conditions, with ad libitum access to food and water, and were acclimatized for 2 weeks before the experiment.

All animal procedures were conducted in accordance with the ARRIVE guidelines and the Principles of Laboratory Animal Care and were approved by the Animal Ethics Committee of Shanghai Jiao Tong University (approval no. 20230302).

### 2.2 Study design and treatment allocation

This study was designed as a sequential, stepwise parameter-optimization experiment in a canine prostate model. The experimental procedure comprised three phases: (1) preliminary assessment of pulse delivery tolerance and selection of candidate voltage ranges; (2) comparison of single-pulse and compound-pulse delivery strategies; and (3) systematic evaluation of electric-field strength, electrode spacing, and electrode insertion depth. Because both prostate lobes of each Beagle were available for intervention, treatment configurations were assigned at the prostate-lobe level to reduce the number of animals required. The left and right prostate lobes were designated as L and R, respectively. Except for the preliminary pulse-tolerance assessment and needle-insertion controls, each predefined parameter configuration was evaluated in three prostate lobes. The complete allocation of prostate lobes and treatment parameters is presented in **Table 1**.

**Table 1:** Experimental animal groups and treatment parameters for compound steep-pulse irreversible electroporation.

Experimental procedure	Animal lateral leaf number	Needle spacing (mm)	Needle depth (mm)	Voltage (V)	Electric field strength (V/cm)	Pulse width (μs)
Determination of the safe ablation voltage	A01-L	10	5	500-2500	500-2500	5
	A01-R	10	5	500-1500	500-1500	50
Determination of the ablation pattern	A02-L, A02-R	10	5	0	0	0
	A03-L, A03-R	10	5	1500	1500	5
	A04-L, A04-R	10	5	1000	1000	50
	A05-L, A05-R	10	5	1500&1000	1500&1000	5&50
Determination of optimal ablation parameters	B01-L	18	10	0	0	0
	B01-R	14	10	0	0	0
	B02-L	10	10	0	0	0
	B02-R, B03-L, B03-R	10	10	1000&500	1000&500	5&50
	B04-L, B04-R, B05-L	10	10	1500&1000	1500&1000	5&50
	B05-R, B06-L, B06-R	10	10	2000&1500	2000&1500	5&50
	B07-L, B07-R, B08-L	14	10	1400&700	1000&500	5&50
	B08-R, B09-L, B09-R	14	10	2100&1400	1500&1000	5&50
	B10-L, B10-R, B11-L	14	10	2800&2100	2000&1500	5&50
	B11-R, B12-L, B12-R	18	10	1800&900	1000&500	5&50
	B13-L, B13-R, B14-L	18	10	2700&1800	1500&1000	5&50
	B14-R, B15-L, B15-R	10	20	1500&1000	1500&1000	5&50

1) The electric-field strengths (V/cm) represent nominal field settings calculated based on the applied voltage and electrode spacing, rather than direct measurements of the electric field distribution within tissue. 2) Because the field strength is 2000&1500 and the needle spacing=18 mm, the required voltage value is larger, due to the limitation of the voltage performance of this instrument, therefore, for the needle spacing=18mm, the field strength 2000&1500 V/cm parameter is not set. 3) “R”, right lobe; “L”, left lobe.

No formal *a priori* sample size or power calculation was performed because the primary objective of this study was exploratory parameter optimization rather than confirmation of a predefined primary hypothesis. Sample size was determined pragmatically based on the feasibility of the large-animal model, sequential evaluation of multiple treatment configurations, and the principle of reducing animal use. Allocation of treatment configurations at the prostate-lobe level enabled evaluation of multiple parameters within the same animal cohort while minimizing the number of animals required.

In the first stage, escalating voltages were applied using 5- and 50-μs pulses to preliminarily assess muscle contraction during pulse delivery and establish candidate voltage ranges. In the second stage, under fixed electrode-placement conditions, the ablation effects of 5-μs pulses, 50-μs pulses, and a compound strategy combining both pulse widths were compared (26); needle-insertion controls received electrode insertion without pulse delivery. In the third stage, a predefined parameter matrix was used to evaluate the effects of electric-field strength, interelectrode spacing, and insertion depth on prostate ablation. Three electric-field configurations, three interelectrode spacings, and two insertion depths were included. The high-field configuration at an 18-mm interelectrode spacing was not applied because the required voltage exceeded the maximum output capacity of the pulse generator. Needle-insertion controls were included for each interelectrode spacing.

### 2.3 Operation procedure

The animals were fasted for 12 h before anesthesia. Fifteen minutes before induction, each animal received a subcutaneous injection of atropine sulfate (0.1 mg/kg; Pfizer, China) administered by a veterinarian. Anesthesia was induced by intravenous injection of tiletamine-zolazepam (Zoletil™, 5 mg/kg; Virbac, France) and maintained with 2% isoflurane in oxygen via an endotracheal tube (AbbVie, USA). These dosages were consistent with standard veterinary anesthesia protocols in dogs (27, 28).

After induction of anesthesia, the animals were placed in the supine position on the operating table and connected to an anesthesia machine, cardiac monitor, and triaxial accelerometer. A urinary catheter was inserted through the urethra to empty the bladder and secure the urethra. The lower abdomen was then shaved, disinfected, and surgically prepared. An abdominal incision was made lateral to the penis to expose the prostate.

The electrode needles were connected to the compound steep-pulse therapeutic device (CSPTA, Shanghai Nortion Medical Technology Co., Ltd., Shanghai, China), and a test pulse was delivered in saline to verify device function. The electrodes were subsequently inserted into the designated prostate lobe(s), and pulse delivery was performed according to the predefined treatment allocation and parameter configurations shown in **Table 1**. For needle-insertion controls, the electrodes were inserted at the designated spacing and depth without pulse delivery.

After the procedure, the electrodes were withdrawn and the surgical incision was closed. The animals were allowed to recover from anesthesia and received prophylactic antibiotics to prevent postoperative infection. They were subsequently housed in the experimental animal facility under aseptic conditions.

### 2.4 Observation indicators

#### 2.4.1 Routine blood, blood biochemistry and urine routine

All dogs were subjected to general and gross observations, blood routine, blood biochemistry and urine examination before sampling on preoperative and postoperative days 7, respectively. In addition, adverse events that occurred during the trial were recorded.

Blood samples were collected from the cephalic vein into vacuum tubes containing K_2_EDTA (for complete blood count) or clot activator (for serum biochemistry). Complete blood count (CBC) was analyzed using a Mindray BC-30Vet veterinary blood cell analyzer (Mindray Bio-Medical Electronics Co., Ltd., Shenzhen, China). Serum biochemistry parameters were analyzed using a Rayto Chemray 240 automatic biochemistry analyzer (Rayto Life and Analytical Sciences Co., Ltd., Shenzhen, China). Urine samples were obtained via free catch mid-stream or cystocentesis. Urinalysis was performed using URIT 11G urine reagent strips and a URIT-180 urine analyzer (both from Guilin URIT Medical Electronic Co., Ltd., Guilin, China). All samples were processed and analyzed within 2 hours of collection.

All procedures followed the manufacturers’ operational guidelines. The analyzers were calibrated daily, and quality control was performed using commercial control sera and urine to ensure accuracy and precision.

#### 2.4.2 Histopathology

On day 7 after the procedure, all dogs were euthanized by intravenous administration of an overdose of sodium pentobarbital (150 mg/kg). Death was confirmed by the absence of a palpable heartbeat and corneal reflex. The prostate tissues were subsequently collected for histopathological analysis.

The tissue specimens were fixed in 10% formalin for at least 24 h and routinely processed for paraffin embedding. Five to ten consecutive sections were prepared perpendicular to the electrode needle tract. Sections with intact tissue morphology, uniform thickness, and no apparent sectioning artifacts were selected for hematoxylin and eosin (H&E) staining (29). Predefined exclusion criteria included incomplete sectioning due to tissue-processing artifacts, electrode placement deviating by more than 2 mm from the intended trajectory, or severe tissue folding or tearing that precluded reliable area measurement. Histopathological changes were examined under a light microscope (Olympus BX53, China).

The ablation zone was identified on H&E-stained sections based on the boundary between coagulative necrosis and adjacent viable prostatic tissue. Digital whole-slide images were analyzed using KFBIO digital pathology software (KFBIO, Ningbo, China). The boundary of the ablation zone was manually delineated, and the ablation area was calculated by the software. Measurements were performed on the evaluable H&E-stained section exhibiting the largest ablation area. The pathologist performing the measurements was blinded to the ablation parameters. A second pathologist independently reviewed a randomly selected subset of measurements, and discrepancies were resolved by joint review and consensus.

#### 2.4.3 Assessment of muscle contraction intensity

Muscle contraction intensity was assessed in real-time during pulse delivery by two independent, trained researchers present in the procedure room. Both observers were blinded to the specific pulse parameter combinations used for each ablation to minimize scoring bias. Because the primary objective was to compare the relative muscle contraction induced by different pulse parameters rather than to quantify neuromuscular activation, a predefined semi-quantitative visual grading scale (I, II, III, IV) was used. Scores were assigned as follows: I indicated no detectable contraction in the limbs or torso; II corresponded to slight, localized flickering or twitching near the electrode site without joint movement; III represented distinct, single contractions causing visible flexion or extension of a single joint with low amplitude; and IV indicated intense, coordinated contractions involving the entire limb or multiple joints, resulting in vigorous gross movement.

### 2.5 Statistical analysis

The statistical analysis of this study was generated by the software R4.2.0 and SAS9.4. Continuous variables were statistically analyzed by ANOVA or t-test when they met the normality condition, and by the Kruskal-Wallis test or Wilcoxon rank-sum test when they did not meet the normality condition. *Post hoc* two-by-two comparisons were corrected for P values using the Bonferroni method.

## 3 Results

### 3.1 General and gross observations

None of the test dogs exhibited any abnormal behaviors related to eating, drinking, defecating, or any other behavior, and the wounds showed no signs of redness, swelling, abnormal blood leaking, or other complications following the procedure. Throughout the observation cycle, all animals’ body weight and temperature were within the normal physiological range with minor fluctuations. Gross observations showed that all test dogs had normal prostate size, color and texture, without anomalies such as tissue adhesion, edema, or fluid buildup (**Figure 1**). All collected prostate specimens met the predefined quality criteria, and no specimens were excluded from the subsequent histopathological analyses.

**Figure 1:**
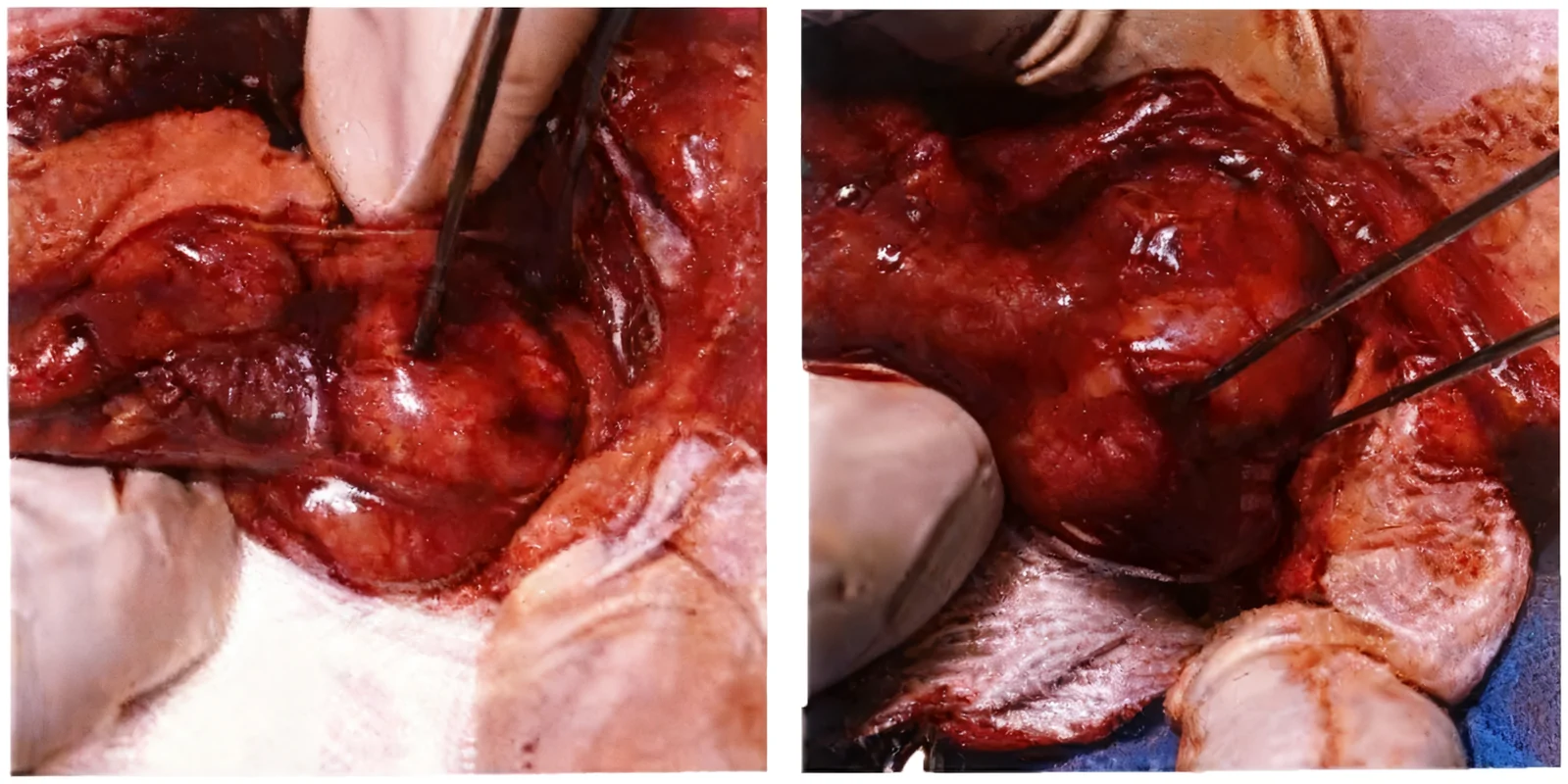
Gross observation of the prostatic ablation sites was performed intraoperatively in beagle dogs.

### 3.2 Determination of the safe ablation voltage

The high-voltage pulse generates an action potential that causes strong muscle shaky. In this study, we observed the muscle shaking of the animals and graded them (I, II, III, IV, the larger the value means the more powerful the shaking) to record the degree of muscle shaking of each animal under different ablation parameters. The specific parameter settings and the degree of muscle shaky are shown in **Table 2**. The results show that under the given pulse width: When the pulse width is 5 μs, the voltage ranges from 500 V to 2500 V; When the pulse width is 50 μs, the voltage ranges from 500 V to 1500 V, all of the muscle contraction strength is within the allowable range.

**Table 2:** Degree of muscle shaky.

Needle spacing (mm)	Needle depth (mm)	Voltage (V)	Electric field strength (V/cm)	Pulse width (μs)	Muscle shaky degree
10	5	500	500	5	I
10	5	1500	1500	5	II
10	5	2000	2000	5	III
10	5	2500	2500	5	III
10	5	500	500	50	I
10	5	1000	1000	50	I
10	5	1500	1500	50	II

### 3.3 Determination of the ablation pattern

The ablation area was observed by histopathology to evaluate the ablation effect of different ablation modes. Pathologic results are shown in **Figure 2**. The specific parameter settings and ablation area data are shown in **Table 3**. The A02 (needle insertion only) was no discharge, and no ablation area on the prostate tissue. The ablation areas of test dogs A03 (5 μs, 1500 V/cm), A04 (50 μs, 1000 V/cm), A05 (5 μs, 1500 V/cm & 50 μs, 1000 V/cm) had an ablation area of 39.3 mm^2^, 64.44 mm^2^, and 307.27 mm^2^, respectively. As a result, the “5 μs, 1500 V/cm” and “50 μs, 1000 V/cm” compound pulse width and electric field strength modes exhibited a better ablation effect.

**Figure 2:**
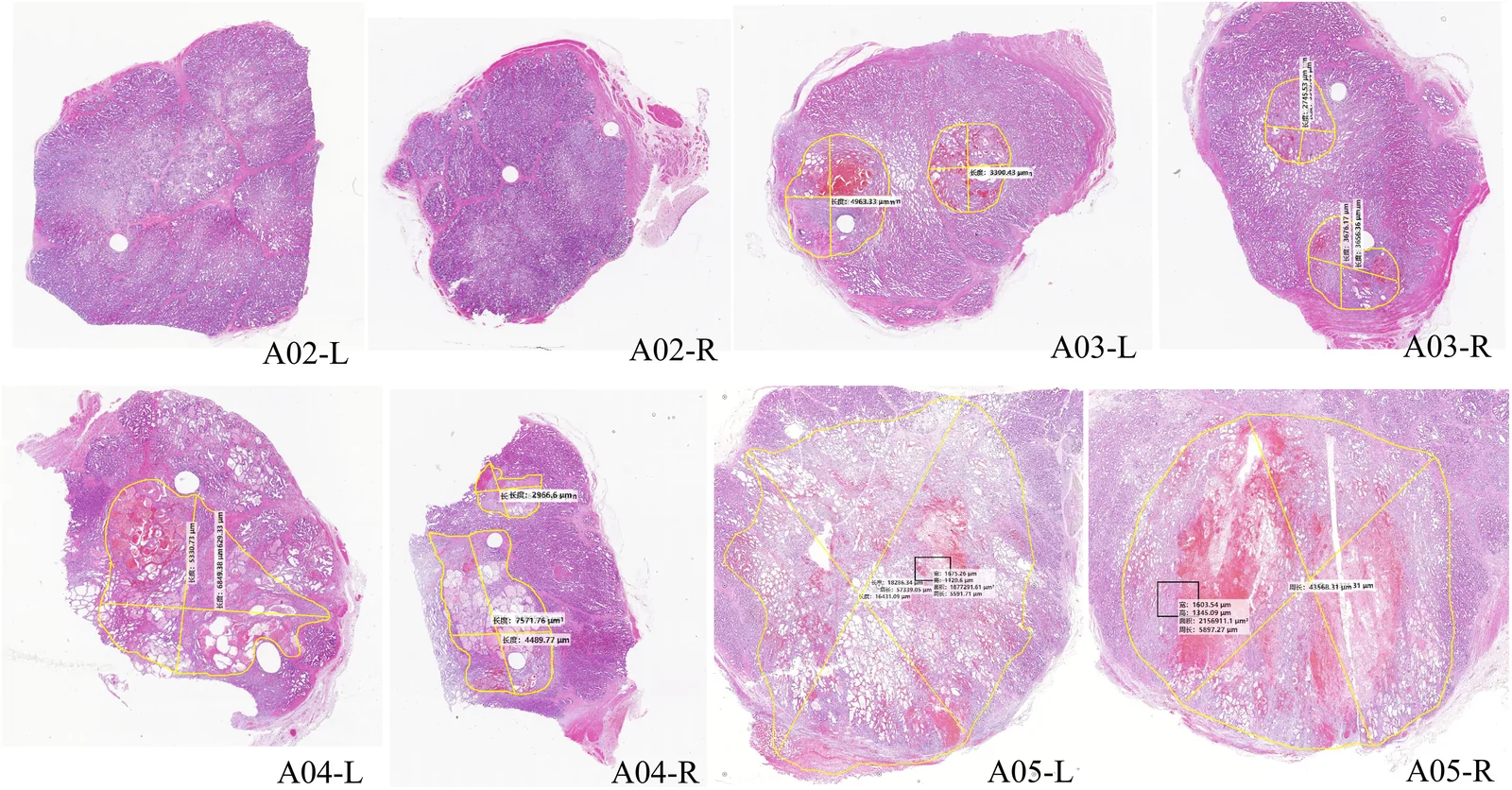
The results of HE staining performed with different ablation pattern at 7 days after ablation.

**Table 3:** Ablation area for different ablation patterns.

Animal number	Needle spacing (mm)	Needle depth (mm)	Voltage (V)	Nominal Electric field strength (V/cm)	Pulse width (μs)	Ablation area (mm^2^)
A02	10	5	0	0	0	0
A03	10	5	1500	1500	5	39.3
A04	10	5	1000	1000	50	64.44
A05	10	5	1500&1000	1500&1000	5&50	307.27

A02 (needle insertion only), A03 (5 μs, 1500 V/cm), A04 (50 μs, 1000 V/cm), A05 (5 μs, 1500 V/cm & 50 μs, 1000 V/cm).

### 3.4 Determination of better ablation parameters

Based on the ablation pattern determined from the above experimental results, different ablation parameters and needle spacing were set (needle depth=10mm). Since the diameter of the prostate gland of Beagle is 2–3 cm above and below, left and right, and anterior and posterior, the needle spacing could not be set more than 20 mm, and in this study, the needle spacing was set at 10, 14, and 18 mm. Define “1000 V/cm, 5 μs &500 V/cm, 50 μs “ as the low parameter group, “1500 V/cm, 5 μs &1000 V/cm, 50 μs “ as the medium parameter group, “2000 V/cm, 5 μs &1500 V/cm, 50 μs” is the high parameter group. Pathologic results are shown in **Figure 3A**.

**Figure 3:**
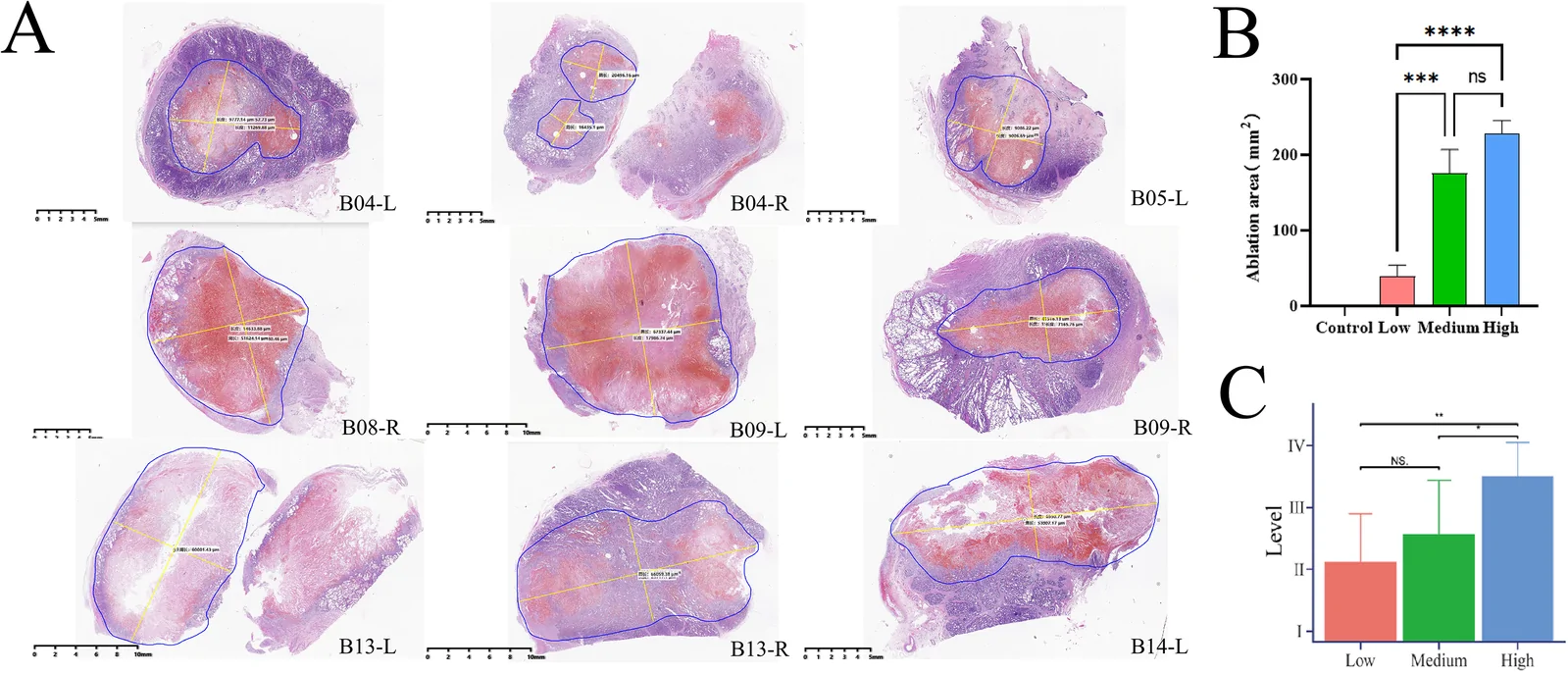
Determination of optimal ablation parameters. **(A)** The results of HE staining performed with the medium parameter group at 7 days after ablation. **(B)** Ablation area under small, medium, and large parameters (*** p<0.001, **** p<0.0001 using ANOVA test; ns indicates no significant difference) **(C)** Muscle shaky degree under small, medium, and large parameters. (* p<0.05, ** p<0.01 using Wilcoxon rank-sum test; ns indicates no significant difference).

The results showed that B01-L, B01-R, B02-L (needle insertion only) had no ablation area and the mean ablation area in the small(n=9), medium(n=9) and large(n=6) parameter test groups was 39.74 ± 43.45 mm^2^, 175.46 ± 94.08 mm^2^, and 228.49 ± 41.26 mm^2^, respectively. Among them, the ablation area in the medium and large parameters was significantly larger than that in the small parameter groups (**Figure 3B**, *p* < 0.05). Still, there was no statistically significant difference in the comparison between the medium and large parameters. The large parameter group exhibited greater animal jitteriness during ablation than the medium parameter group (**Figure 3C**, *p* < 0.05). Therefore, the medium parameter group was the superior ablation parameter.

### 3.5 Ablation area for different needle spacing

To investigate the effect of needle spacing on the ablation effect, we also compared the difference in ablation area between different needle spacing for this parameter. The mean values of ablation area for the needle pitch 10mm, 14mm and 18mm groups were 83.93 ± 25.31mm^2^, 226.25 ± 108.25mm^2^ and 216.20 ± 64.17mm^2^, respectively. The needle spacing 10, 14 and 18 mm groups were able to obtain a certain ablation area, and the 14 mm needle spacing group yielded the largest ablation area among the tested configurations (**Table 4**).

**Table 4:** Multi-group comparison of different needle spacings.

Statistic	10 mm	14 mm	18 mm
N (Missing)	3 (0)	3 (0)	3 (0)
Mean ± SD	83.93 ± 25.31	226.25 ± 108.25	216.20 ± 64.17

### 3.6 Ablation area at different needle depths

Because the Beagle prostate is approximately 2–3 cm in diameter, insertion depths of 10 mm and 20 mm were selected to represent two feasible treatment depths within the gland while avoiding penetration beyond the prostatic capsule. Ablation was performed under the same parameters and the ablation areas of 10mm and 20mm needle depths were compared. The results showed that the ablation area of 10 mm and 20 mm needle depth was 83.93 ± 25.31 mm^2^, 90.10 ± 32.92 mm^2^, respectively (**Table 5**). The ablation area of 10 and 20 mm needle depths could be obtained to a certain extent, and the ablation area of the 20 mm group was slightly larger than that of the 10 mm group.

**Table 5:** Comparison of two groups with different needle depths.

Statistic	10 mm	20 mm
N (Missing)	3 (0)	3 (0)
Mean ± SD	83.93 ± 25.31	90.10 ± 32.92

### 3.7 Routine blood, blood biochemistry and urinalysis

Preoperative and postoperative hematological parameters are summarized in **Table 6**. RBC count, hemoglobin concentration, and platelet count remained within the corresponding reference intervals before and after the procedure. Individual pre-to-postoperative changes in these parameters did not exceed the respective reference change values (RCVs), indicating no clinically relevant alterations beyond expected physiological variation (30).

**Table 6:** Preoperative and postoperative hematological parameters in 20 Beagle dogs compared with published reference intervals.

Parameter	Unit	Pre-operation	Post-operation	Reference interval	RCV reference
RBC	×10^12^/L	6.93 ± 0.60	6.25 ± 0.71	5.57-8.38	17.20%
Hgb	g/L	165.17 ± 12.73	146.57 ± 17.45	134-194	17.50%
Platelets	×10^9^/L	251.17 ± 29.92	321.71 ± 75.09	168-482	39.60%
WBC	×10^9^/L	12.25 ± 3.68	18.07 ± 4.46	5.05-14.76	54.70%

An increase in WBC count was commonly observed after the procedure, with the mean postoperative value exceeding the upper limit of the reported reference interval. However, assessment of individual longitudinal changes showed that the magnitude of WBC variation remained within the RCV of 54.7% in all dogs except A01. Dog A01 showed a postoperative WBC increase exceeding the RCV, consistent with a more pronounced inflammatory response.

## 4 Discussion

The present study evaluated the ablation performance and short-term safety of CSP-IRE for prostate ablation in healthy Beagle dogs. CSP-IRE induced effective prostatic tissue ablation without apparent procedure-related complications during the 7-day observation period. Among the tested conditions, the compound pulse protocol combining 5-μs pulses at 1500 V/cm with 50-μs pulses at 1000 V/cm produced a larger ablation area than the single-pulse protocols. The medium parameter field group showed a favorable balance between ablation area and muscle contraction, while electrode spacing and insertion depth further influenced the ablation area. These findings suggest that integrating different pulse widths and electric-field strengths may enhance ablation performance and warrant further discussion of the potential mechanisms underlying this effect.

A notable finding of the present study was that the compound pulse protocol produced a substantially larger ablation area than either of the single-pulse protocols tested. Similar phenomena have been observed in potato tuber, rabbit liver, mouse tumors, and porcine liver tissues (31–33). One possible explanation proposed in previous studies is that pulses with different durations and electric-field strengths interact with biological tissue in complementary ways, thereby producing more effective membrane permeabilization over a broader population of cells than can be achieved with a single waveform alone (34, 35). Specifically, high-voltage narrow pulses can perforate cell membranes, but these perforations often do not result in cell necrosis. The superimposed low-pressure broad pulse can widen these perforations, thus killing the cancer cells more completely (34). However, the precise mechanisms underlying this synergy remain unclear and were not investigated here. Future work combining tissue electrical characterization with field modeling will be required to elucidate them.

The medium-parameter protocol (1500 V/cm, 5 μs and 1000 V/cm, 50 μs) provided the most favorable balance between ablation efficacy and procedural safety. Although the high-parameter protocol delivered a higher electric field, it did not significantly increase the ablation area. This finding is consistent with the sigmoidal dose-response characteristics of irreversible electroporation, whereby lesion size reaches a plateau once the tissue-specific threshold has been exceeded (36). Since the estimated IRE threshold for canine prostate tissue is approximately 1072 V/cm (37), the medium-parameter protocol likely already operated near this plateau, limiting the benefit of further field escalation. In contrast, increasing the electric field significantly aggravated procedure-related muscle contraction. Because the excitation threshold of nerves and muscles is substantially lower than the threshold required for irreversible electroporation, higher field strengths preferentially enhance neuromuscular stimulation rather than therapeutic efficacy (38). Therefore, further increasing the nominal electric field primarily intensified procedure-related neuromuscular stimulation rather than improving ablation efficacy. Within the range of parameters evaluated in this study, the medium-parameter protocol offered the most favorable balance between ablation efficacy and procedure-related neuromuscular stimulation.

Within the range evaluated in this study, an inter-electrode spacing of 14–18 mm provided effective tissue coverage, whereas increasing needle insertion depth had little influence on the cross-sectional ablation area. Compared with the 10 mm spacing, both the 14 mm and 18 mm configurations produced substantially larger mean ablation areas, consistent with previous experimental and computational studies demonstrating that increasing electrode spacing expands the volume of tissue encompassed by the effective electric field (39). However, despite the greater electrode separation, the 18 mm spacing did not produce a larger ablation area than the 14 mm spacing. This finding suggests that the benefit of increasing electrode spacing is not linear. As the distance between electrodes increases, the electric field strength within the central region gradually decreases, potentially reducing the volume of tissue exposed above the irreversible electroporation threshold (40). Consequently, although the 14 mm spacing yielded the largest mean ablation area, its comparable performance with the 18 mm configuration indicates that the practical electrode spacing for this canine prostate model is likely to lie within the range of 14–18 mm rather than at a single optimal value. In contrast, increasing the insertion depth from 10 mm to 20 mm produced only a modest increase in ablation area, suggesting that, within the anatomical dimensions and treatment depths evaluated, lesion size in the cross-sectional plane was influenced more by electrode spacing than by insertion depth (40, 41).

Postoperative hematological evaluation revealed a transient increase in white blood cell counts, whereas no clinically relevant changes were observed in erythrocytes, hemoglobin, or platelets. Similar transient leukocytosis has been reported after surgical procedures in dogs and is generally considered a physiological response associated with surgical stress, tissue injury, and activation of innate inflammatory pathways rather than a sign of systemic toxicity (24, 42). Given that IRE induces localized cellular injury and subsequent immune activation, the observed WBC elevation may reflect a short-term tissue injury–related inflammatory and immune response to the ablation procedure (43). The absence of abnormalities in other hematological parameters further supports the favorable systemic safety profile of this approach.

## 5 Strength and limitation

The present study adopted a sequential, stepwise experimental design that enabled systematic parameter optimization within a single large-animal model, allocating treatment configurations at the prostate-lobe level to reduce inter-animal variability and animal use. The study further incorporated blinded histopathological assessment, objective evaluation of procedure-related muscle contractions, and short-term safety monitoring. In addition, the compound steep-pulse protocol represents a distinct waveform strategy that represents a waveform strategy that integrates multiple pulse widths and electric-field strengths within a single treatment sequence. Together, these features provide a comprehensive evaluation of CSP-IRE efficacy and safety and support its further translational investigation.

Several limitations should be acknowledged. First, this study was performed in healthy canine prostates, which may not fully reflect the tissue characteristics and electroporation responses of human benign or malignant prostate tissues. Validation in disease-specific models and human-relevant systems is therefore required before clinical translation. Second, as a sequential parameter-optimization study, the relatively small number of prostate lobes evaluated for each condition and the variability in ablation area limited definitive assessment of parameter differences and interactions. Larger studies with multifactorial designs are needed for further validation. Third, the reported electric-field strengths represent device output settings rather than the actual tissue electric field distribution. Moreover, mechanistic interpretations of compound-pulse effects were primarily based on studies in non-prostatic tissues and require confirmation in prostate-specific models using approaches such as impedance measurement and computational modeling. Finally, although the histological sections preserved the urethral structure, systematic evaluation of adjacent critical structures, functional outcomes, and quantitative neuromuscular responses was not performed. Although needle insertion-only controls accounted for mechanical effects associated with electrode placement, they could not fully exclude potential thermal or systemic procedural responses unrelated to electroporation. Future studies incorporating anatomical mapping, sham stimulation controls, longer follow-up, and electromyography are warranted to further characterize the safety profile of CSP-IRE.

## 6 Conclusion

In the canine prostate model, the compound pulse protocol demonstrated superior ablation efficacy over single-pulse protocols. The medium-parameter protocol (1500 V/cm, 5 μs and 1000 V/cm, 50 μs) achieved a favorable balance between ablation area and procedure-related neuromuscular stimulation. An electrode spacing of 14–18 mm provided effective tissue coverage, whereas needle insertion depth showed limited influence on lesion size. No serious adverse events were observed, supporting the safety and feasibility of CSP-IRE for prostate ablation.

## Data Availability

The original contributions presented in the study are included in the article/supplementary material. Further inquiries can be directed to the corresponding author.
